# Malaria treatment policies and drug efficacy in Haiti from 1955-2012

**DOI:** 10.1186/2052-3211-6-10

**Published:** 2013-11-11

**Authors:** Michael E von Fricken, Thomas A Weppelmann, Jennifer D Hosford, Alexander Existe, Bernard A Okech

**Affiliations:** 1 Department of Environmental and Global Health, University of Florida, P.O. Box 100188, Gainesville, FL 32610, USA; 2 Emerging Pathogens Institute, University of Florida, P.O. Box 100009, Gainesville, FL 32610, USA; 3 National Laboratory of Public Health, Ministry of Public Health and Sanitation, Angle Delmas 33 et Rue Charbonnieres, Haiti

**Keywords:** Hispaniola, Haiti, Malaria treatment policy, Chemotherapy, Chloroquine, Anti-malarial drug resistance, *Plasmodium falciparum*, Pyrimethamine

## Abstract

**Objectives:**

Chloroquine (CQ), after 67 years of use in Haiti, is still part of the official treatment policy for malaria. Several countries around the world have used CQ in the past due to its low incidence of adverse events, therapeutic efficacy, and affordability, but were forced to switch treatment policy due to the development of widespread CQ resistance. The purpose of this paper was to compile literature on malaria treatment policies and antimalarial drug efficacy in Haiti over 67-year period.

**Methods:**

A systematic review of PubMed, Web of Science, and the Armed Forces Pest Management Board, was conducted to find pertinent documents on national malaria treatment policies and antimalarial drug efficacy studies in Haiti between 1955 and 2012. A total of 329 citations and abstracts were reviewed independently by two researchers, of which thirty three met the final inclusion criteria of studies occurring in Haiti between 1955 and 2012 which specifically discuss malaria treatment policies and drug efficacy.

**Results:**

Results suggest that CQ has been the predominant antimalarial drug in use from 1955 to 2012. In 2010 single dose primaquine (PQ) was added to the national treatment policy, however it is not clear whether this new policy has been put into practice.

**Conclusions:**

Although no widespread CQ resistance has been reported, some studies have detected low levels of CQ resistance. Increased surveillance and monitoring for CQ resistance should be implemented in Haiti.

## Introduction

Malaria persists in Hispaniola despite its elimination from other Caribbean countries [1]. It’s estimated that over 99% of malaria cases in Haiti are caused by *Plasmodium falciparum* with *Anopheles albimanus* serving as the principal mosquito vector [2-4]. The burden of malaria is high in Haiti, relative to its population of 10 million people, with 80% of the Haitian population living in areas where malaria is endemic [5,6]. Historically, Haiti has under-reported the number of malaria cases, largely due to limited resources, inadequate surveillance and a shortage of trained personnel [7-9]. The antimalarial chloroquine (CQ) has been relied on heavily in Haiti, due to its low incidence of adverse events, affordability, and perceived therapeutic efficacy, despite the emergence of CQ resistance globally.

To better understand Haiti’s prolonged commitment to CQ, we compiled reports and publications on malaria chemotherapeutic policies and antimalarial resistance in Haiti from 1955 to 2012 based on a systematic search of historical literature. Prior to 1955, quinine was used intermittently during periods of US occupation [10]. The purpose of this paper is to document the history of Haiti’s malaria treatment policies, while providing a useful reference for antimalarial drug resistance studies in Haiti.

## Methods

### Data sources

Published studies and reports about malaria in Haiti were identified from an electronic search of MEDLINE®/PubMed® (1955-2012) and Web of Science (1955-2012). A search of gray literature was carried out on the Armed Forces Pest Management Board (AFPMB) database [11], which contains publications and reports from the Centers for Disease Control and Prevention (CDC), World Health Organization (WHO), and Pan American Health Organization (PAHO). Search terms were chosen to capture malaria treatment policy and drug efficacy studies in Haiti only, using a combination of simple subject headings and term combinations, focusing on treatment, malaria, and Haiti.

### Study selection

The selection criteria of the data sources included are; 1) reports from the period of 1955 to 2012, 2) all studies carried out in Haiti, 3) and studies that discuss malaria. Full text manuscripts and all citations which met the predefined selection criteria were obtained and examined by two independent researchers. Final inclusion and exclusion decisions were made with 100% agreement between the examiners based on studies that reported malaria treatment policies and drug efficacy for final inclusion. Where duplications were observed the most recent version of the manuscript was selected. The year 1955 was used as a start date for analysis, because it coincided with the Global Malaria Elimination Program campaign in Haiti.

## Results

A total of 329 citations and abstracts in the electronic searches were found. Of these, 85 citations and abstracts met the preliminary criteria and their full texts were reviewed. Thirty three studies described malaria treatment policies or antimalarial drug efficacy in Haiti from 1955-2012 [1,2,4,6-9,12-37].

### Characteristics of included studies

**Figure 1:**
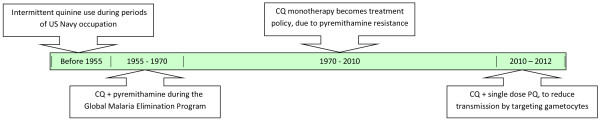
Timeline of Haitian malaria treatment policies.

Chloroquine was first introduced in Haiti in 1955, when The Eighth World Health Assembly recommended its use in combination with pyrimethamine (P) for the elimination of malaria. The chloroquine/pyrimethamine (CQ/P) combination strategy was the first treatment policy switch in Haiti replacing the previous accepted practice of quinine for the treatment of malaria [38-40]. According to the data sources, from 1955 to 1970, CQ/P was administered in a prophylactic manner through mass drug administration (MDA) campaigns, as part of the Global Malaria Eradication Program in Haiti [13,15,16,40]. From 1970 to 2010, the treatment for malaria switched from a combination therapy of CQ/P therapy to CQ monotherapy for uncomplicated malaria cases [16]. In 2010 a CDC report mentioned the addition of the transmission blocking drug primaquine (PQ) at a single dose of 0.75 mg/kg, suggesting a major change in policy [36]. All final articles (N = 33) that we evaluated reported CQ as or part of the standard treatment policy in Haiti. A brief summary of treatment policies can be seen in the timeline provided (Figure 1).

### Evidence for treatment resistance in Haiti

Only fourteen studies from the data sources examined [6,9,12-19,21,26,32,35] discussed chemotherapeutic efficacy or patient treatment outcome data (Table 1). Six of these fourteen studies contained documentation of resistance to CQ or P [6,16,18,19,21,32]. The first year of reported of anti-malarial resistance, where *P. falciparum* had documented pyrimethamine resistance was 1971 [16]. In 1982, a report of possible resistance to CQ treatment was suggested [6] after a patient on CQ monotherapy had resurgence in parasitemia on day 28 of treatment. A follow-up *in vitro* test found 4/16 *P. falciparum* cultures including the *P. falciparum* strains from the patient with resurgent parasitemia, required CQ doses large enough that suggested possible resistant *P. falciparum* parasites. This report provided the first credible *in vitro* evidence of *P. falciparum* resistance to CQ in Haiti [18]. In 1984, an *in vivo* and *in vitro* study, tested for *P. falciparum* parasite resistance to sulfadoxine/pyrimethamine (S/P) [19] to provide baseline data on susceptibility in the event that CQ resistance developed in Haiti. The results indicated parasite resistance to pyrimethamine alone, but not to sulfadoxine. Despite *in vitro* evidence of resistance to pyrimethamine, all *in vivo* infections were susceptible to a combination of S/P [19]. In 1985, another *in vivo* study to complement the previous *in vitro* assay found resistance to pyrimethamine alone [21]. More recently, a report by Londono et al, has documented the detection of genetic markers via PCR, for CQ resistance in five of 79 patients (6%) from the Artibonite Valley [32]. However, a study by Neuberger et al, found zero of 49 infected patients to have mutations suggestive of CQ resistance [36]. These studies present conflicting evidence about the presence or absence of CQ drug resistance in the populations studied, with no definitive documentation of *in vivo* resistance reported.

**Table 1:** Reports of treatment efficacy for malaria in Haiti

**Period**	**Sample size**	**Site in Haiti**	**Drug combination**	**Source of data used**	**Resistant**	**Susceptible**	**Reference**
1960-1966	N/A	Country Wide	CQ/P	Secondary	N/A	CQ	PAHO. 1967 [14]
1961	N/A	Country Wide	CQ	Secondary	N/A	CQ	WHO. 1965 [13]
1962-1965	N/A	Country Wide	CQ/P	Secondary	N/A	CQ/P	Mason J and Philippe A, 1967 [12]
1962-1965	N/A	Petitie Goave	CQ & CQ/P	Secondary	N/A	CQ/P	WHO, 1968 [15]
1971	N/A	Country Wide	CQ	Secondary	VV, P & CG	CQ	WHO, 1972 [16]
1976	N/A	Country Wide	N/A	Secondary	N/A	CQ	WHO, 1978 [17]
1980	N/A	Country Wide	CQ	Secondary	N/A	VV CQ	PAHO, 1980 [9]
1981-1983	92	Les Cayes, Port-au-Prince, Limbe, Gros-Morne and Jacmel	CQ	Secondary	VT & VV CQ	CQ	Duverseau YT, Magloire R, et al., 1986 [6]
1982	19	Port-au-Prince	CQ	Secondary	VT & VV CQ	N/A	Magloire R and Nguyen-dinh P, 1983 [18]
1982	18	Port-au-Prince	P & S/P	Primary	VT & VV P	VV S/P	Nguyen-Dinh P, Zevallos-Ipenza A, et al., 1984 [19]
1985	22	Port-au-Prince	P & S/P	Primary	VT P	VV & VT S/P	Nguyen-Dinh P, Payne D, et al., 1985 [21]
1995	N/A	Country Wide	N/A	Primary	N/A	VV CQ	Drabick JJ, Gambel JM, et al., 1997 [26]
2006-2007	79	Artibonite Valley	CQ	Secondary	PCR CQ	N/A	Londono BL, Eisele TP, et al., 2009 [32]
2010-2011	49	Leogane	CQ	Primary	N/A	PCR CQ	Neuberger Zhong K, et al., 2012 [36]

Key: Chloroquine (CQ), pyrimethamine (P), chloroquine with pyrimethamine (CQ/P),sulfadoxine with pyrimethamine (S/P), *in vivo* susceptibility (VV), *in vitro* susceptibility (VT), DNA examined for mutations via polymerase chain reaction (PCR).

## Discussion

Following the widespread use of CQ/P from 1955-1968, resistance to pyrimethamine was reported in Haiti in 1971 [16]. It was suggested that parasite adaptation to pyrimethamine had occurred due to selective drug pressure from the MDA campaign, which ended in 1968, resulting in its removal from the national treatment policy [16,19].

Haiti continued to rely predominantly on CQ as part of the treatment for malaria in Haiti up until 2010, when single dose PQ was added to the official policy for the treatment of uncomplicated malaria in Haiti [2,37]. Haiti represents a unique scenario where PQ has not been used previously on the island due to the absence of *Plasmodium vivax,* but is being introduced to specifically block malaria transmission by targeting the adult stage *Plasmodium falciparum* parasites. It remains unclear whether or not PQ has been implemented in practice, nor have any studies examined population rates of G6PD deficiency in Haiti, a genetic mutation that places deficient individuals at risk of acute hemolytic anemia when exposed to PQ.

Overall, we found the use of CQ has featured prominently in the management of malaria in Haiti for decades. While CQ resistance in many South and Central American countries has forced these countries to change policies, the Haitian Ministry of Health (MSPP) has continued to rely almost entirely on CQ as the principal treatment for malaria. Despite such prolonged use, our results found no conclusive evidence of the presence or absence of CQ resistance in Haiti (Table 1). However, findings from the studies that report on treatment sensitivity are lacking due to small sample sizes [6,18,19,21,32,36], localized enrollment [6,15,18,19,21,32,36], and missing information on patient treatment outcomes, significantly limiting their ability to influence national treatment policies (Table 1). Ten of the fourteen studies that make mention of drug efficacy, relied solely on secondary sources of data, often only containing brief comments on treatment effectiveness, which further suggest an absence of designed drug sensitivity studies occurring in Haiti [6,9,12-18,32]. The two most recent studies examining CQ resistance by Londono and Neuberger gave different results about the presence of genetic markers of CQ resistance in Haiti [32,36]. However, both studies had small sample sizes of 79 and 48 respectively and were located in different regions of the country. CQ resistance mutations are emerging in Haiti, but to what extent remains unknown, suggesting a need for increased surveillance for parasite resistance.

### Limitations

We were unable to examine gray literature from the MSPP as most documents were lost during the 2010 earthquake. Although we excluded non-English databases, we believe this omission to not be very significant, since we relied heavily on WHO and PAHO reports, which are based on both English and non-English databases and reports. There were limited data pertaining to treatment failures and parasite resistance, in addition to an overall lack of reporting between 1985-2005 due to political unrest [8,32].

## Conclusions

As of 2012, the few documented reports of CQ resistance in Haiti did not exceed the WHO recommended threshold treatment failure rate of ≥ 10% [41]. However, due to limited surveillance on drug efficacy and barriers to patient follow up, further studies are necessary to determine if rates of CQ resistance fall below this threshold in Haiti. Meanwhile, Non-government organizations, foreign aid agencies, and the Haitian MSPP should consider implementing a comprehensive malaria control program while CQ remains a viable treatment option [32,35,36]. Other drug regimens, such as artemisinin-based combination therapies, are part of an arsenal of available treatment options, in the event of CQ resistance. The affordable cost of CQ, and its’ low incidence of adverse events make it an ideal treatment option on a large scale. It is our recommendation that increased surveillance and monitoring for CQ resistance be implemented, due to significant gaps in data on CQ treatment failure and resistance in Haiti. Regarding the recent addition of PQ to the national policy, more information is needed on how PQ is tolerated in this population, given the absence of information on G6PD prevalence rates, and whether or not this policy has been put into practice in Haiti. Future studies examining transmission rates in Haiti may generate valuable information on the impact single dose PQ has on malaria rates, potentially providing a template for elimination in other low transmission settings globally.

## Abbreviations

CQ: Chloroquine; P: Pyrimethamine; CQ/P: Combined chloroquine-pyrimethamine; PQ: Primaquine; S: Sulfadoxine; S/P: Combined sulfadoxine-pyrimethamine; ACT: Artemisinin-based combination therapy; WHO: World Health Organization; PAHO: Pan American Health Organization; MSPP: Ministry of Health and Population; MDA: Mass Drug Administration.

## Competing interests

The authors declare that they have no competing interests.
